# Cutaneous microvascular function is reduced in winter compared with summer in healthy young adults

**DOI:** 10.14814/phy2.70784

**Published:** 2026-02-19

**Authors:** James F. Bangle, William E. Jennings, Alyssa J. Guadagni, Georgia R. Albino, Andrew J. Grundstein, S. Tony Wolf

**Affiliations:** ^1^ Department of Kinesiology University of Georgia Athens Georgia USA; ^2^ Department of Geography University of Georgia Athens Georgia USA

**Keywords:** endothelium, nitric oxide, seasonal variation, temperature, vitamin D

## Abstract

Seasonal differences in temperature and ultraviolet radiation (UVR) exposure may alter NO‐mediated vasodilation in the cutaneous microvasculature. We assessed seasonal variation in cutaneous microvascular function in 10 healthy (23 ± 2; 6 men, 4 women) adults in the summer/early‐fall and winter/early‐spring. Microvascular (intradermal microdialysis and local skin heating [42°C]) endothelial function was assessed at two visits separated by ~6 months. Mean daytime (sunrise to sunset) outdoor temperature and 2‐h peak UV Index were recorded for the 30 days prior to each experimental visit. Serum vitamin D concentrations [25(OH)D] were measured for each visit. Multivariate regression analysis evaluated the factors (temperature, UV Index, and [25(OH)D]) that best explained variance in microvascular endothelial function. The local heating response (74.37 ± 12.21 %CVC_max_ vs. 55.89 ± 15.50 %CVC_max_, *p* = 0.009) and the NO contribution to that response (61.73% ± 9.67% vs. 38.13% ± 13.26%, *p* = 0.004) were lower in winter/early‐spring compared to summer/early‐fall. The best‐fit regression model suggested that 30‐day average outdoor temperature and 2‐h peak UV Index were positively and negatively associated with NO‐dependent cutaneous vasodilation, respectively. Healthy adults exhibit lower NO‐mediated cutaneous vasodilation in the winter/early‐spring compared with summer/early‐fall, which is partially explained by seasonal differences in outdoor temperature.

## 1 INTRODUCTION

The full expression of cutaneous vasodilation is dependent upon endothelium‐derived nitric oxide (NO) (Bruning et al., 2012), and the function of the cutaneous microvasculature is reflective of generalized vascular function (Debbabi et al., 2010; Kenney, 2017). As such, any physiological input that influences NO production and bioavailability may alter cutaneous microvascular function. Environmental exposures represent a subset of physiological stressors that may have multifactorial impacts on NO‐mediated cutaneous vasodilation. Air temperature and ultraviolet radiation (UVR) significantly vary across seasons in temperate regions, and both of these environmental factors may influence cutaneous microvascular function.

Evidence for the effects of UVR exposure on cutaneous microvascular function is mixed. We have previously demonstrated that acute UVR exposure (UVB and broad‐spectrum UVR) reduces endothelial NO‐mediated cutaneous vasodilation in healthy young adults, secondary to local increases in reactive oxygen species production and/or degradation of bioactive folate (5‐methyltetrahydrofolate [5‐MTHF]; an eNOS cofactor) (Wolf et al., 2018, 2019). Conversely, acute exposure to ultraviolet A (UVA) may increase liberation of NO stores independent of endothelial NO synthase (eNOS) (Liu et al., 2014); however, the potential role of this mechanism in vascular function has not been explored. The effects of chronic UV exposure on NO‐mediated cutaneous vasodilation are even less well understood. Using regional variation in skin pigmentation (i.e., more or less tanned/sun exposed skin) as a proxy for seasonal UVR exposure, we demonstrated that within‐limb variation in skin pigmentation did not alter NO‐mediated cutaneous vasodilation (Fisher et al., 2023). Participants in that study were tested at a single timepoint at the end of summer (August to October); thus, it remains unclear whether seasonal variations in UVR exposure may influence cutaneous microvascular function.

Seasonal changes in UVR exposure may also influence endothelial function through changes in vitamin D status. Exposure to UVB induces cutaneous vitamin D synthesis (Wolf & Kenney, 2019), which may play a role in NO bioavailability. Mechanistically, the bioactive metabolite of vitamin D, calcitriol, may improve NO production and bioavailability by (1) upregulating the transcription of eNOS, (2) reducing oxidative stress via inhibition of NADPH oxidase and/or increased expression of superoxide dismutase, and/or (3) reducing inflammation by inhibiting inflammatory factors such as nuclear factor‐κB (NF‐κB) (Andrukhova et al., 2014; Wolf & Kenney, 2019). Serum [25(OH)D] is directly related to NO‐mediated cutaneous vasodilation in young adults with a wide range of skin pigmentation, which may suggest that adequate UVB‐induced vitamin D production promotes optimal function of the cutaneous microvasculature (Wolf et al., 2022).

The potential impact of seasonal temperature variations on cutaneous microvascular function is similarly unclear. A large multisite study across the United States demonstrated an inverse correlation between temperature and blood pressure (that study demonstrated similar associations between UVR and blood pressure); however, the effects on endothelial function were not examined (Weller et al., 2020). Reduced brachial artery flow‐mediated dilation (FMD) responses have been demonstrated in the winter compared with the summer (Haliloğlu et al., 2016; Honda et al., 2021; Iwata et al., 2012; Widlansky et al., 2007), and temperature (Widlansky et al., 2007) and serum vitamin D concentrations (Haliloğlu et al., 2016) were associated with those seasonal changes. Widlansky, et al. (Widlansky et al., 2007) examined the post‐occlusive reactive hyperemia response – which is often reported as an index of microvascular function – and found that reactive hyperemia was associated with outdoor and room temperature, although there was no significant difference between seasons. Importantly, the reactive hyperemia response is predominantly mediated via non‐NO mechanisms (Crecelius et al., 2013; Wong et al., 2003). To our knowledge, potential seasonal changes in NO‐mediated microvascular function have yet to be investigated. Additionally, it is unclear whether seasonal changes in vascular function are (1) mediated by changes in NO production and bioavailability, or (2) related to changes in temperature, UVR, and/or vitamin D status.

Thus, the primary purpose of this study was to evaluate seasonal differences in NO‐mediated cutaneous vasodilation responses in healthy young adults. We hypothesized that NO‐mediated cutaneous vasodilation would be greater in the summer and early‐fall months compared with the winter and early‐spring. An additional, exploratory aim was to determine the relative contributions of temperature, UVR, and circulating vitamin D concentrations on NO‐mediated cutaneous vasodilation.

## 2 METHODS

### 2.1 Participants

All experimental protocols were approved by the Institutional Review Board at the University of Georgia. Written and verbal consents were obtained from all subjects prior to participation, according to the Declaration of Helsinki. All participants underwent an initial screening that included a health history questionnaire and measures of height, weight, and BP. Ten healthy individuals (6 men, 4 women) aged 19–27 years completed experimental visits in both seasons (winter/early‐spring and summer/early‐fall). Participants were counterbalanced for the order of season in which they were tested. Participants were recreationally active (i.e., not student‐athletes), non‐hypertensive (systolic BP [SBP] <130 and diastolic BP [DBP] <80 mmHg), non‐obese (BMI <30 kg/m^2^), nonsmokers who were free of cardiovascular disease, kidney disease, skin disease, pigmentation disorders or skin allergies, and were not taking any medications that have known vascular effects. Participants were not excluded from taking vitamins or supplements. Women had regular menstrual cycles (*n* = 1) or were taking oral contraceptives (*n* = 3). A urine pregnancy test confirmed the absence of pregnancy before experimental visits. To increase generalizability of our findings, and based on data demonstrating negligible impacts (Williams et al., 2020; Williams & MacDonald, 2021), women were tested without regard to menstrual cycle or oral contraceptive phase.

### 2.2 Experimental procedures

Data were collected from July 2024 to September 2025 in a thermoneutral (~23°C) laboratory. Summer/early‐fall was defined as June–October and winter/early‐spring as January–April. These timepoints were selected because there are significant differences in temperature in the US and adults have lower serum vitamin D levels in the winter and early‐spring compared to the summer and early‐fall (Godar et al., 2011). Furthermore, serum vitamin D [25(OH)D] has a relatively long half‐life of 15 days; therefore, the time between October and January allowed sufficient time for [25(OH)D] to significantly decline (Institute of Medicine (US) Committee to Review Dietary Reference Intakes for Vitamin D and Calcium, 2025). Participants were fasted, had not consumed caffeine for at least 8 h, and refrained from vigorous exercise for at least 24 h prior to testing. All testing was performed in the morning hours between 8:00 am and 12:00 pm.

### 2.3 Intradermal microdialysis

With the participant in a semi supine position, one intradermal microdialysis fiber (10 mm, 55‐kDa cutoff membrane; CMA, Kista, Sweden) was placed in the dermal layer of the ventral aspect of the left forearm for local delivery of pharmacological agents. Pharmacological agents were mixed before use, dissolved in lactated Ringer's solution, sterilized using syringe microfilters (Acrodisc; Pall, Port Washington, NY), and wrapped in foil to prevent degradation due to light exposure. Solutions were perfused through the microdialysis fiber at a rate of 2 μL/min (Bee Hive controller and Baby Bee microinfusion pumps; Bioanalytical Systems) (Bruning et al., 2012). Local red blood cell flux was measured directly over the microdialysis site via an integrated laser‐Doppler flowmetry probe placed in a local heating unit (Moor Instruments SHO2, Moor Instruments, Inc., Wilmington, DE). After placing the microdialysis fibers, a ~60‐min period was allowed for resolution of the hyperemia response associated with needle placement. After the resolution of hyperemia, baseline data (~20 min) were collected (PowerLab/LabChart, ADInstruments, Bella Vista, NSW, Australia) before starting a standard local heating (42°C) protocol, during which lactated Ringer's solution was perfused through the microdialysis fiber (Minson et al., 2001). The local heating response is characterized by an initial axon reflex‐mediated peak, followed by a brief nadir and then a gradual rise to an elevated blood flow plateau. After observing a local heating plateau, 15 mM of NG‐nitro‐l‐arginine methyl ester (L‐NAME; NO synthase inhibitor; Sigma‐Aldrich, St. Louis, MO; Cat. No. N5501) was perfused, allowing for quantification of NO‐dependent vasodilation (Wolf et al., 2024). Following the observation of a stable L‐NAME plateau, 28 mM sodium nitroprusside (SNP; Sigma‐Aldrich, St. Louis, MO; Cat. No. 71778) was perfused and local temperature was increased to 43°C to elicit maximal vasodilation.

Mean arterial pressure (MAP) was calculated for each phase of the local heating protocol using BP from an automated BP monitor (CONNEX Spot Monitor, Hill‐Rom, Chicago, IL). For each phase of the local heating protocol, cutaneous vascular conductance was calculated as laser‐Doppler flux divided by MAP and expressed as a percentage of maximum (%CVC_max_) (Minson et al., 2001). Flux values for the baseline, local heating plateau, and L‐NAME plateau phases were taken as 5‐min average values. Because the initial peak and oftentimes the maximal response are relatively transient, flux data were taken as the average value over a ~30‐s period for those phases. The NO contribution to cutaneous vasodilation was calculated as the difference between the local heating and L‐NAME plateau responses (∆%CVC_max_) (Wolf et al., 2024; Wong & Hayat, 2025).

### 2.4 Meteorological data

Meteorological data were extracted (weatherSTEM.com) for each of the 30 days prior to each participant's experimental visit. Hourly daytime (sunrise to sunset) ambient air temperature and UV Index were recorded using the closest WeatherSTEM station to the participant's location. Average daytime temperature and 2‐h peak UV Index were used in our analyses to accurately reflect seasonal variations in temperature and UVR intensity from the sun.

### 2.5 Assessment of serum vitamin D and skin pigmentation

Blood samples were collected in serum separator tubes during each visit. Serum was isolated via centrifugation and stored at −80°C for future analysis. Serum [25(OH)D], the primary circulating metabolite of vitamin D, was quantified in triplicate using an ELISA kit according to the manufacturer's instructions (CrystalChem, Elk Grove Village, IL; Cat. No. 80987).

Skin pigmentation was measured by skin reflectance spectrophotometry (DermaSpectrometer; Cortex Technology, Hadsund, Denmark) to determine the melanin index (M‐index) of the skin on the forehead and the dorsal aspect of the forearm. These sites were chosen because they are relatively well‐exposed to sunlight; thus, increased M‐index (i.e., darker skin pigmentation) at these sites may act as a proxy for seasonal differences in UV exposure. M‐index values are reported as the average of the two sites.

### 2.6 Statistical approach

Statistical analyses were performed with GraphPad Prism 10.5.0 (GraphPad Software, San Diego, CA) and SPSS 29.0.1.0 (IBM, Armonk, NY). A two‐way ANOVA was used to assess the effects of season (winter/early‐spring vs. summer/early‐fall) and phase (baseline, peak, local heating plateau, L‐NAME plateau, and ∆%CVC_max_) for the local heating protocol. Post hoc comparisons with Tukey's corrections were performed for specific planned comparisons. A paired two‐tailed *t*‐test was used to compare CVC_max_ between seasons. Paired one‐tail *t*‐tests were used to compare temperature, 2‐h peak UV Index, and serum [25(OH)D] between seasons. Due to the difficulty of estimating an effect size because no studies have previously examined seasonal variation in NO‐mediated cutaneous microvascular function, we did not perform an a priori power analysis. However, we have included Hedges' *g* effect sizes where comparisons were statistically significant. Conservatively assuming an effect size of g = 1.32, based on the seasonal difference in the local heating plateau response, we determined that a sample size of *n* = 10 yields a power of 1‐β = 0.95.

To evaluate the relative influence of temperature, 2‐h peak UV Index, and serum [25(OH)D] on seasonal variation in ∆%CVC_max_, we conducted ordinary least squares regression analyses with all possible combinations of the three predictors. To identify the most parsimonious model explaining variation in ∆%CVC_max_, each model was evaluated using Akaike Information Criterion (AIC), which balances model fit and complexity. The model with the lowest AIC was selected as the best‐fitting model. Absence of multicollinearity was confirmed with variance inflation factor (VIF) < 5.0 for all variables.

## 3 RESULTS

Subject characteristics are presented in Table 1. Anthropometric characteristics and blood pressures were typical for this age group. Neither systolic (Summer, 110 ± 9 vs. Winter, 112 ± 10; *p* = 0.49) nor diastolic (Summer, 68 ± 6 vs. Winter, 68 ± 7; *p* = 0.17) blood pressure differed between seasons.

**TABLE 1:** Subject characteristics.

	Mean ± SD	Range
Age, years	23 ± 2	19–27
BMI, kg/m^2^	23 ± 2	20–28
Systolic BP, mmHg	110 ± 9	98–122
Diastolic BP, mmHg	68 ± 6	60–76

*Note*: Values are means ± SD. *n* = 10 (6 men and 4 women).

Abbreviations: BMI, body mass index; BP, blood pressure.

### 3.1 Seasonal differences in endothelial function

**FIGURE 1:**
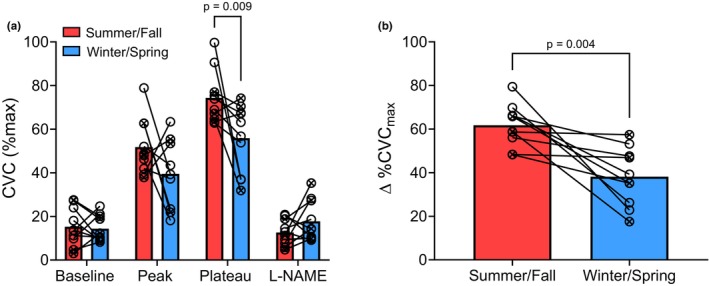
Seasonal comparisons in (a) cutaneous vascular conductance (%CVC_max_) during each phase of the local heating protocol and (b) the nitric oxide contribution to the local heating plateau (∆%CVCmax). Male and female participants (*n* = 10; 6 M, 4 F) are represented by circles with and without an X through them, respectively. Two‐way ANOVA was used to evaluate effects of season and phase of the local heating protocol.

Seasonal comparisons in cutaneous microvascular responses at each phase of the local heating protocol are depicted in Figure 1. There was a main effect of local heating phase [F (2.370, 41.87) = 133.1; *p* < 0.0001], but not season [F (1, 18) = 3.42; *p* = 0.08], and there was a significant interaction between local heating phase and season [F (2.370, 41.87) = 6.12; *p* = 0.003]. There were no differences in baseline (Summer, 15.27 ± 8.77 %CVC_max_ vs. Winter, 14.39 ± 6.09 %CVC_max_; *p* = 0.80), the initial axon reflex‐mediated peak (Summer, 51.82 ± 12.80 %CVC_max_ vs. Winter, 39.52 ± 16.06 %CVC_max_; *p* = 0.09), or the L‐NAME plateau (Summer, 12.64 ± 5.89 %CVC_max_ vs. Winter, 17.76 ± 9.32 %CVC_max_; *p* = 0.16) (Figure 1a). The magnitude of the local heating plateau was significantly diminished in the winter/early‐spring compared with summer/early‐fall (74.37 ± 12.21 %CVC_max_ vs. 55.89 ± 15.50 %CVC_max_, *p* = 0.009, *g* = 1.32; Figure 1a). Likewise, the NO contribution to the local heating response (∆%CVC_max_; Figure 1b) was attenuated in winter/early‐spring compared with summer/early‐fall (61.73 ± 9.67% vs. 38.13 ± 13.26%, *p* = 0.004, *g* = 2.03; Figure 1b). There were no differences in maximal CVC values between seasons (Summer, 2.56 ± 0.92 flux/mmHg vs. Winter, 2.24 ± 0.76 flux/mmHg; *p* = 0.43).

### 3.2 Potential contributors to seasonal variation in microvascular endothelial function

Meteorological, vitamin D, and M‐Index data for each season are presented in Table 2. Daytime temperature (*p* = 0.0003, *g* = 2.94) and 2‐h peak UV Index (*p* = 0.04, g = 1.13) were significantly lower in the winter/early‐spring compared with summer/early‐fall. Serum [25(OH)D] was significantly reduced in the winter/early‐spring compared to summer/early‐fall (*p* = 0.02, *g* = 0.28). Likewise, M‐Index was marginally but significantly lower in winter/early‐spring compared with summer/early‐fall (*p* = 0.002, *g* = 1.07).

**TABLE 2:** Seasonal comparisons in temperature, ultraviolet radiation intensity, vitamin D, and M‐index.

	Summer/early‐fall	Winter/early‐spring
Daytime temperature (°C)	25.67 ± 2.36	13.86 ± 5.16*
2‐h Peak UV Index (0–11+)	4.50 ± 1.09	2.93 ± 1.64*
[25(OH)D] (ng/mL)	45.50 ± 14.47	41.45 ± 14.10*
M‐index (a.u.)	34.63 ± 2.49	32.08 ± 2.25*

*Note*: Values are means ± SD. UV, ultraviolet; [25(OH)D]; serum vitamin D concentrations; M‐index, a reflectance‐based measure of skin melanization.

* *p* < 0.05 compared with summer/early‐fall.

Temperature was independently associated with in ∆%CVC_max_ (*p* = 0.01), but 2‐h peak UV Index and [25(OH)D] were not (*p* ≥ 0.10). However, the best‐fit model (AIC = 159.07) explaining the variance in ∆%CVC_max_ included temperature and 2‐h peak UV Index (Table 3), but not serum [25(OH)D]. In the multivariate model, temperature was positively associated with ∆%CVC_max_ and explained 33% of the variance, whereas 2‐h peak UVR was negatively associated with ∆%CVC_max_ and explained 15% of the variance.

**TABLE 3:** Results of the multivariate regression analysis describing the variables that significantly contribute to seasonal variation in NO‐mediated cutaneous vasodilation.

Variable	Standardized coefficient	*t*	*p*	*R* ^2^	Adjusted *R* ^2^	Partial *R* ^2^
Regression	‐	‐	0.00	0.38	0.31	‐
Temperature	2.42	2.91	0.01	‐	‐	0.33
UV Index	−6.62	−1.74	0.10	‐	‐	0.15

Abbreviation: UV, ultraviolet.

## 4 DISCUSSION

The primary aim of this study was to determine if healthy young adults exhibit seasonal variations in cutaneous microvascular endothelial function. Our data suggest that NO‐mediated cutaneous vasodilation is lower in the winter/early spring compared with summer/early fall. A secondary aim was to assess the relative contributions of outdoor temperature and UVR, and circulating [25(OH)D], to the observed seasonal variation in cutaneous microvascular function. We found that average outdoor temperature in the 30 days preceding experimental visits was the only variable that was independently associated with NO‐dependent cutaneous vasodilation; however, the best‐fit model explaining the variance in NO‐mediated vasodilation included 30‐day average temperature (positive association) and 2‐h peak UV Index (negative association), whereas circulating [25(OH)D] was excluded from the model.

We have previously demonstrated that acute UVR exposure reduced NO‐dependent cutaneous vasodilation via increased oxidative stress and/or degradation of 5‐MTHF (Wolf et al., 2018, 2019). We subsequently showed that there was no effect of within‐limb variation in skin pigmentation (a proxy for regional variation in seasonal UVR exposure) on NO‐dependent cutaneous vasodilation at the end of summer (Fisher et al., 2023). Furthermore, we have demonstrated a direct relation between circulating [25(OH)D] (driven by UVB‐induced cutaneous vitamin D synthesis) and NO‐mediated cutaneous vasodilation (Wolf et al., 2022) in healthy young adults with a wide range of skin pigmentation. Considering the findings of those studies, it is unclear whether chronic UVR exposure may (positively or negatively) influence cutaneous microvascular function. The negative association between 2‐h peak UVR and NO‐mediated cutaneous vasodilation in this study, along with no effect of circulating [25(OH)D], may be explained by the fact that participants in this study were vitamin D sufficient in both seasons. Of note, most of the participants in our previous study (conducted in Centre County, PA) were vitamin D deficient (<20 ng/mL; *n* = 10) or insufficient (21–30 ng/mL; *n* = 17), with only 6 of the participants being in the sufficient range (>30 ng/mL) (Wolf et al., 2022). In contrast, 76% of values in the current study (conducted in Athens‐Clarke County, GA – a region with less seasonal variation in UVB intensity compared with the previous study) were sufficient and there were no participants who were vitamin D deficient. Collectively, these findings may suggest there is an optimal balance of UVR exposure; that is, UVR exposure beyond that which is required to ensure vitamin D sufficiency may negatively affect cutaneous microvascular function via increased oxidative stress or degradation of 5‐MTHF, resulting in reduced NO production and bioavailability. Importantly, the participants in this study were all lightly‐to‐moderately pigmented; future studies are needed in a more diverse cohort to determine whether skin pigmentation modulates the effects of seasonal variations in UVR intensity.

Our study demonstrated that outdoor temperature was positively associated with NO‐mediated cutaneous vasodilation. We are unable to discern whether the observed effects are due to improved endothelial function associated with higher temperatures (e.g., a heat acclimatization effect), poorer endothelial function associated with lower temperatures, or a combination of both. Passive heat therapy consistently improves cutaneous microvascular (Brunt et al., 2016) and conduit artery function (Ely et al., 2019; Imamura et al., 2001; Kihara et al., 2002; Ruiz‐Pick et al., 2025). Mechanistically, increased shear stress associated with heat‐induced increases in peripheral blood flow elicits an upregulation in eNOS expression, thus improving NO production and bioavailability (Green et al., 2017; Hambrecht et al., 2003). Indeed, demonstrated that the increases in blood flow and shear stress are obligatory in inducing endothelial adaptations (Green et al., 2010). Conversely, chronic exposure to colder temperatures may be partially responsible for reduced NO‐mediated cutaneous vasodilation. For example, 5 weeks of moderate cold exposure in rats increased circulating endothelin (ET)‐1, a potent vasoconstrictor, and increased expression of ET type A receptors (responsible for ET‐1‐induced vasoconstriction) in the heart and renal cortex (Chen & Sun, 2006). These potential mechanisms warrant future investigation in the context of seasonal variation in vascular endothelial function.

Other work has demonstrated seasonal variation in conduit artery endothelial function (FMD) in older populations and/or those with cardiometabolic diseases, with most studies showing reduced function in winter compared with the summer (Haliloğlu et al., 2016; Honda et al., 2021; Iwata et al., 2012; Widlansky et al., 2007). Although FMD is predominantly mediated by NO, other mechanisms including the release of prostaglandins and endothelium‐derived hyperpolarizing factors may explain a substantial portion of the response (Green et al., 2014). Our findings agree with those studies demonstrating lower FMD values in the winter compared with the summer and extend those findings by directly quantifying changes in NO‐mediated vasodilation (Wolf et al., 2024). Our data suggest that poorer endothelial function in winter compared with the summer is explained by reduced NO bioavailability.

In regions with seasonal variation, higher blood pressures and increased risk for cardiovascular‐related events are evident in the wintertime (Fares, 2013). Endothelial dysfunction typically precedes the development of hypertension and increases cardiovascular risk (Hadi et al., 2005; Heitzer et al., 2001; Noon et al., 1997; Taddei et al., 1992). Although the current investigation is limited to the cutaneous microvasculature, this model reflects generalized (e.g., conduit arteries, the renal circulation, coronary arteries, etc.) endothelial function/dysfunction, and cutaneous microvascular dysfunction often precedes dysfunction in other circulatory beds (Debbabi et al., 2010; Kenney, 2017). Thus, consideration of these data, alongside previous data demonstrating reduced FMD in winter compared with summer (Haliloğlu et al., 2016; Honda et al., 2021; Iwata et al., 2012; Widlansky et al., 2007), may provide evidence for a role of relative endothelial dysfunction – characterized by reduced NO bioavailability – in wintertime increases in cardiovascular risk.

## 5 LIMITATIONS

A limitation of this study is the use of data from weather stations to assess the contributions of environmental exposures to seasonal variation in endothelial function. Although we used nearby weather stations to assess ambient air temperatures and UVR intensity in the weeks leading up to each experimental visit, these conditions only represent the potential exposures at the individual level – not actual exposures. An analysis of the National Health and Examination Survey (NHANES) from 2009 to 2012 found that 44% of adults spent 30 min or less outdoors on workdays and 20% reported 30 min or less outdoors on non‐work days (Beyer et al., 2018). Therefore, the significant amount of time spent indoors may reduce the potential effects of outdoor temperature and UVR, and we have no way in the current study to assess actual exposures at the individual level. Future work should look to use personal ambient air temperature and UVR monitoring to better understand the relation between these environmental factors and vascular function.

Another potential limitation is that neither physical activity (PA) levels nor cardiovascular fitness were tested in this study. PA levels are typically higher in the summer compared to other seasons, and moderate‐to‐vigorous PA is greater in the summer compared to the winter (Garriga et al., 2022; Turrisi et al., 2021). The evidence is somewhat unclear regarding whether regular exercise training or habitual physical activity improves endothelial function in healthy young adults without evidence of impaired vascular function (Clarkson et al., 1999; DeSouza et al., 2000; Moe et al., 2005; Rognmo et al., 2008; Tinken et al., 2008). However, aerobic exercise training improves NO‐mediated endothelial function in older adults (Black et al., 2008; DeSouza et al., 2000) – the population who is most likely to experience wintertime increases in cardiovascular risk. Seasonal changes in physical activity patterns at the individual level are an important area of future inquiry in relation to seasonal changes in vascular endothelial function and cardiovascular risk, particularly in older adults and other at‐risk populations.

Finally, as previously noted, we are unable to determine whether the seasonal differences here are due to improved endothelial function associated with warmer temperatures, poorer endothelial function associated with colder temperatures, or a combination of both. Notably, NO‐mediated dilation for the global data set (winter/early‐spring and summer/early‐fall combined; ~50%) is in line with previous reports in healthy young adults (typically 50%–60%) (Fisher et al., 2023; Halstead et al., 2023; Kim et al., 2018; Wolf et al., 2019), with markedly lower values in the winter compared to typical average values. This study is a first step in showing seasonal changes in NO‐dependent microvascular vasodilation and will inform future studies that will compare vascular endothelial function in summer and winter relative to more temperate seasons (spring and fall).

## 6 CONCLUSIONS

In summary, this is the first study to our knowledge to demonstrate seasonal variations in NO‐mediated cutaneous vasodilation and its relation with outdoor temperature and UV intensity. These findings align with previous work showing similar seasonality of conduit artery function, blood pressure, and cardiovascular risk, and may provide mechanistic support for a role of NO in those seasonal differences. An additional implication of our findings relates to study design; cross‐sectional or interventional studies should consider the timing of testing so that groups, interventions, etc. are balanced for time of year to eliminate potential confounding effects of season. These data provide early evidence toward the development of lifestyle interventions to mitigate seasonal differences in vascular endothelial function and cardiovascular risk.

## AUTHOR CONTRIBUTIONS

The authors' responsibilities were as follows: James F. Bangle, Alyssa J. Guadagni, and S. Tony Wolf designed research; James F. Bangle, William E. Jennings, Alyssa J. Guadagni, and Georgia R. Albino conducted research; James F. Bangle and S. Tony Wolf interpreted results of experiments; James F. Bangle and S. Tony Wolf analyzed data; James F. Bangle drafted manuscript; James F. Bangle, William E. Jennings, Alyssa J. Guadagni, Georgia R. Albino, and S. Tony Wolf revised, edited, and approved final version of manuscript.

## FUNDING INFORMATION

No external funding was received for this research.

## CONFLICT OF INTEREST STATEMENT

The authors declare no conflicts of interest.

## Data Availability

Data will be made available upon reasonable request.
